# Effects of Turmeric Concentrate on Cardiovascular Risk Factors and Exercise-Induced Oxidative Stress in Healthy Volunteers; an Exploratory Study

**DOI:** 10.34172/apb.2023.052

**Published:** 2022-07-02

**Authors:** Maha Noordin Abu Hajleh, Emad Abdol Sahib Al-Dujaili

**Affiliations:** ^1^Department of Cosmetic Science, Pharmacological and Diagnostic Research Centre, Faculty of Allied Medical Sciences, AlAhliyya Amman University, Zip code (19328), Amman, Jordan.; ^2^Centre for Cardiovascular Science, Queen’s Medical Research Institute, University of Edinburgh, Edinburgh, Scotland, UK.

**Keywords:** Turmeric extract, Curcumin, Antioxidants, Blood pressure, Cardiovascular disease, Oxidative stress, Lipid peroxidation

## Abstract

**Purpose:**

Evidence suggests that turmeric intake can improve antioxidant defense, blood pressure (BP), ageing and gut microbiota. The effects of turmeric concentrate (curcumin) intake on cardiovascular risk factors and exercise induced oxidative stress were investigated.

**Methods:**

A randomized placebo-controlled study was performed to assess the effects of turmeric extract in healthy volunteers before and after a 30 min exercise bout. Participants (n=22) were given either turmeric concentrate or placebo supplements. Anthropometry, BP, pulse wave velocity (PWV), biomarkers of oxidative stress, perceived exertion and lipid peroxidation were assessed.

**Results:**

In the turmeric group, the expected BP response to exercise following turmeric was blunted and the increase was not significant compared to basal values followed by a decrease in final BP and PWV values. There were no significant differences in all baseline parameters between the placebo and the curcumin groups (*P*>0.05). A significant increase was observed in urinary antioxidant power (*P*=0.031) and total polyphenol levels (*P*=0.022) post turmeric intervention. The distance ran by the participants taking turmeric was significantly longer (*P*=0.005) compared to basal value. Those who took the placebo did not show significant changes.

**Conclusion:**

Our study suggests that turmeric concentrate intake can reduce BP and improve antioxidant, anti-inflammatory status and arterial compliance. Turmeric may improve exercise performance and ameliorates oxidative stress. Larger studies are warranted to validate these findings and test more cardiovascular risk factors.

## Introduction

 Regular physical exercise is known to convey several health benefits and considered to be good practice to combat many metabolic diseases including cardiovascular disease (CVD), cancer, obesity, and diabetes.^1-3^ Paradoxically, it has become evident over the last 30 years that despite beneficial effects of exercise, various types of exercise including eccentric muscle contraction, aerobic exercise induced muscle damage (EIMD), strenuous and prolonged exercise generate inflammatory cytokines and reactive oxygen species (ROS).^4,5^ For example, EIMD triggers inflammatory responses and promotes the production of transcription factors such as nuclear factor kappa B (NF-κB) through the production of ROS.^3,6^ It has been reported that the negative effects associated with eccentric exercise such as inflammation and delayed onset muscle soreness (DOMS) are caused by a large increase in inflammatory cytokines in the working muscle, plasma and brain generated as a result of oxidative stress which stimulates the production of free radicals.^7,8^ Retamoso et al^9^ found that eccentric exercise may cause DOMS causing discomfort of skeletal muscles, and following strenuous exercise, a considerable generation of ROS was observed which resulted in muscle damage.^8,10^ However, physical exercise was found to promote alterations in skeletal muscle physiology by modifying gene transcription and protein synthesis.^11^ Exercise induces the production of reactive ROS which activates endogenous antioxidant defense mechanisms.^12,13^ and stimulates cytokine production “myokines” from skeletal muscles. This appears to have important anti-inflammatory, metabolic and physiological roles. Increased levels of ROS during exercise play a pivotal role in the regulation of cell signaling and contribute to muscle adaptation after physical exercise by regulating cell signaling via myokines which represent a significant link between the contracting muscle and metabolic homeostasis.^14^

 The byproducts of aerobic mitochondrial metabolism, ROS are essential for activating cellular repair or apoptosis. However, enhanced levels of ROS are associated with inflammation, cellular stress, cancer and CVD and can initiate the development of hypertension via numerous effects that are not fully understood.^15,16^ They mediate pathological alterations in the kidney, central nervous system and vasculature resulting in the onset of chronic hypertension and stimulate adaptive immunity which can contribute to hypertension by interrelating with these organs.^17^ To counter the increase of ROS, the increased action of antioxidants defense systems will reduce the net cellular ROS load and scavenging ROS reduces the blood pressure (BP).^18^ Moreover, ROS oxidize numerous biological macromolecules such as proteins, lipids, and nucleic acids causing structural and functional changes in these substances. The production of eicosanoids derives from the oxidation of polyunsaturated fatty acids which act as an important signaling intermediates in modulating cellular physiological function via regulating various transcription factors.^19^ Lipid oxidation produces hydroperoxides that generate a broad range of reactive intermediates, such as prostaglandin F2α (isomer F2-isoprostanes) and malondialdehyde (MDA).^20-22^ Both molecules analyzed to indicate lipid peroxidation levels in biological fluids, however MDA is the most used biomarker of oxidative stress in several conditions such as cancer, chronic obstructive pulmonary disease, and CVDs.^23^

 In hypertensive patients, the incidence of oxidative stress can be determined in biological fluids by the thiobarbituric acid assay.^23,24^ These patients also display impaired antioxidant defense mechanisms and have low ferric-reducing ability of plasma (FRAP) which links to both systolic BP (SBP) and diastolic BP (DBP).^18^ It is vital to monitor both SBP and DBP throughout exercise. In addition, pulse wave velocity (PWV) is regarded the gold standard measurement of arterial stiffness and it is generally assessed by using carotid-femoral or brachial-ankle approaches.^25^ PWV rises with stiffening of the aorta and consequently causes an earlier return of reflected pressure waves from the periphery to the aorta which enhances aortic SBP and reduces DBP.^25^ Thus, PWV measurement may be used in the prognosis of CVDs.^26^ Oxidative stress induces an increase in BP by multiple mechanisms, stimulating increased ROS and oxidative damage.^27^ Factors such as angiotensin II (Ang II), endothelin-1 (ET-1), aldosterone (Aldo), and salt can induce activation of NADPH oxidases (Noxs) that generate ROS which influence multiple systems involved in the pathophysiology of hypertension. In the heart, the production of ROS exceeds the capacity of the antioxidant defense mechanism to buffering the ROS resulting in cardiac dysfunction, ischemia-reperfusion injury, hypertrophy, cell death, and heart failure.^28^ Indeed, hypertension can lead to the development of CVD due to the increased production of oxidants, low antioxidant capacity in the vasculature and decreased nitric oxide (NO) bioavailability.^27^ Previously, Rodrigo and colleagues have reported an increase in systemic BP and peripheral vascular resistance due to the inhibition of NO synthesis and marked increase in oxidative stress.^29^ To prevent this, it is vital to maintain the oxidant: antioxidant balance to protect the body against exercise induced oxidative stress.^29^

 Turmeric rich in curcumin (multi-potent molecule) can strengthen the antioxidant defense due to its numerous putative therapeutic properties. Turmeric is a natural polyphenol derived from the rhizome of *Curcuma longa*^28,30^ and has antioxidative, anti-inflammatory, cardiovascular, mental wellbeing and ageing protective effects, and interacts with gut microbiota and several other actions.^31-34^ Turmeric extract have been shown to improve oxidative stress markers by acting on cytokine/ROS-mediated inflammatory pathways to reduce the expression of NF-**κ**B/cyclooxygenase-2 (COX-2), enhancing antioxidants activity and inhibiting the production of prostaglandin and NF-kB signaling.^35-37^ In addition, turmeric may have musculo-protective effects against EIMD by inhibiting free radical formation in injured skeletal muscles.^36,38,39^ Turmeric may have direct antihypertensive effects possibly due to its antioxidant, anti-inflammatory, Ca^2+^ ions interference, α_2_-adrenergic receptor stimulation, and renin-angiotensin system inhibition.^40,41^ The phenol rings of curcumin can scavenge the potent oxidant peroxynitrite (ONOO - ) generated by the interaction of superoxide (O^−2^) and NO and the reactive hydroxyl radical (OH - ).^42,43^ The aim of this short preliminary study was to investigate the effects of turmeric extract (rich in curcumin) intake on CVD risk factors (BP, PWV and lipid peroxidation), and exercise induced oxidative stress in human volunteers.

## Materials and Methods

###  Subject recruitment

**Figure 1:**
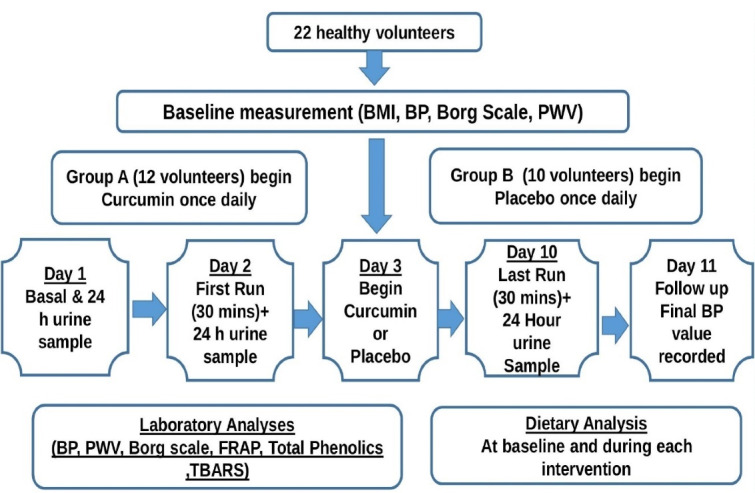
Study protocol demonstrating the trial arrangements and measurements performed for all participants

 Twenty-two healthy volunteers were recruited among staff and students at Queen Margaret University (QMU). Participants recruited were both male (n = 10) and female (n = 12). An inclusion/exclusion criterion was established. Healthy, non-smokers, normotensive subjects who fell between the ages of 19-60 years were eligible to take part in the study. The exclusion criteria were those who had any history of previous or current medical health conditions such as Diabetes, Urinary Tract Infections, hypertension, and asthma. Pregnant women and patients on any other medication such as anti-inflammatory drugs or contraceptives were also excluded (see Figure 1). All participants were asked to limit their daily alcohol intake during the study with no alcohol being allowed within 24 hours of any testing period. They were also asked to refrain from foods which may contain turmeric, fruits, and vegetables rich in polyphenols. Participants were also asked not to take part in any intense physical activity during the study to avoid any chance of errors occurring in our results. All research took place in the QMU gym and labs. Prior to the study, an in-formation sheet was handed out to participants who wished to take part, highlighting the aims of the research and what was expected of them. As part of the Ethics guidelines, participants were asked to sign a formal written consent form to confirm they were happy with the information provided and they were eligible to withdraw from the study at any time. A simple health questionnaire was completed by the participants, which allowed us to assess their current state of health, to ensure they were fit to take part, before we were able to begin testing.

###  Supplement

 Subjects were randomly divided into two intervention groups, turmeric (n = 12) and placebo (n = 10). Each participant in both groups was supplied with 8 capsules, either turmeric concentrate (Curcumin C3 Complex-500 mg, Jarrow Formulas, Los Angeles, CA, USA) or placebo capsules made by filling empty gelatin capsules with corn flour. The gelatin capsules were purchased from Amazon and were suitable for vegetarians. We termed the turmeric extract group as such or simply as curcumin intervention. Supplements were to be taken once daily with lunch, for 8 days within the 11-day study period.

###  Study design

 We have adopted a randomized placebo-controlled parallel study to investigate the efficacy of curcumin supplementation on exercise-induced oxidative stress and BP over a period of 11 days. Each subject was given 3 urine bottles for the collection of their urine samples over a 24 hours period within the study and was asked to run for a 30 minutes period on a treadmill at their own desired speed before and after intervention and the distance travelled was recorded each time. Figure 1 demonstrates the trial arrangement and measurements performed for each participant.

###  Anthropometry and physiological measurements

 Measurements ofbody mass index (BMI) was facilitated by recording** t**he weight and height of each subject day 1 of the study allowing to calculate their BMI using the equation: [BMI = Weight (kg)/height (m)^2^]. The assessment was performed to limit any risk of oxidative stress occurring through the presence of inflammation in subjects who may be slightly overweight.

 Dietary Intake assessments were completed using a 24 hours diet diary on day 2 and compared to those obtained on day 10 of the study to determine the total energy, carbohydrate, fat, and protein levels of the participants. All data was processed through WinDiets software (WinDiets, 2015; Robert Gordon University, Aberdeen, UK). Diet diary was important to assess whether the administration of turmeric supplementation had any impact on the participants total energy intake.

####  Non-invasive cardiovascular risk factors

 BP was measured as follows: On day 1, basal BP readings recorded; on day 2, BP detections were recorded before and after the 30 minutes first run; on day 10, BP detections were recorded before and after the 30 min final run and final BP detections were recorded on day 11. The subjects were usually seated for 10 minutes in a relaxed room before their BP was measured using an electronic sphygmomanometer. The average of 3 BP detections was calculated to increase reliability and minimize inaccurate readings due to stress and the “white coat” effect.

 Arterial compliance measured by PWV was performed between the carotid and femoral artery (PWVcf) by means of a validated Vicorder^TM^ device (Skidmore Medical Limited, Bristol, UK). This was performed by measuring the distance between the midpoints of two oscillometric cuffs, placed at the collar (carotid artery) and at the proximal right femur (femoral artery). The mean PWVcf of three measurements was recorded and the data expressed in m/s.^25^

####  Perceived exertion

 A Borg rating of perceived exertion scale (1-10) was used to measure each subject’s level of exertion and intensity during each of the 30 minutes runs.^44^

###  Urinary analysis 

 All 24 hours urine samples collected were weighed on a scale and 2 aliquots of each urine sample was labelled (date, subject code, and net weight) and stored at -20°C until assayed. All urine samples were kept in a freezer until all 66 samples were collected for final analysis. Three biomarkers were used to analyze our samples including FRAP, total phenolics and TBARS. The frozen samples were left to defrost completely prior to the analysis and an aliquot was diluted with distilled water, if necessary, to match the standards used. Samples were assayed in triplicates to minimize any errors and the average was obtained.

####  TBARS assay

 Thiobarbituric acid reactive substances (TBARS) were used to determine the concentration of MDA (byproduct of lipid peroxidation) which is a major indicator of cellular injury and thus considered to be an oxidative stress marker. A modification of previous methods was used to monitor and estimate TBARS in the urine samples.^45^ In brief, 0.1 mL of urine sample, 0.2 mL of Tris buffer and 0.1 mL of Ascorbic acid was added to the incubation tube. After 15 minutes of incubation at 37°C, 0.4 mL of TCA and 0.8 mL of TBA solution were added into the tubes. Tubes were then incubated at 99°C for 15 minutes and centrifuged at 4000 rpm for 10 minutes. The absorbance of the samples was read at 532 nm. The data were calculated from standard curve constructed simultaneously. The standard curve concentrations (100, 50, 25, 10, 2.5 and 0.625 μmole/L) were prepared from the stock MDA standard (1 mmole) in distilled water.

####  Antioxidant power assay

 FRAP (Ferric ion reducing antioxidant power) assay was used and modified to determine the antioxidant concentration present in the urine samples as presented by Benzie and Strain.^46^ The antioxidants present in the urine sample reduce ferric-2,4,6-tri-2-pyridyl-s-triazine (Fe^3+^ TPTZ) to the ferrous form (Fe^2+^) under acidic conditions. As a result, an intense blue/purple color is detected with maxi-mum absorbance at 593 nm. Excess Fe^3+^ is used therefore as the rate limiting phase of Fe^2+^ TPTZ production and hence color formation. Each sample was placed in the spectrometer, the FRAP working reagent was added with a 4 min waiting period before the absorbance concentration results were recorded. The results were then used to calculate the antioxidant concentration of the urine by comparing it to a standard curve. This was previously prepared using known concentrations of ferrous sulphate (FeSO_4_) solution (zero-1.0 mmole/L). The results to determine the antioxidant concentration were recorded as a mean concentration of Fe^2+^ produced (mmole/day) taking the total 24 hours urine in consideration.

####  Total phenols method

 The concentration of total phenolic compounds in the urine samples were determined.^47^ Briefly, 50uLof each sample was added to 2.5 mL of Folin Ciocalteu reagent and 1.7 mL of sodium carbonate (Na_2_CO_3_) solution was added, and the reaction mixture was left to develop (in the dark) for 2 hours. A spectrophotometer was used to measure the absorbance of the solution at 765 nm against water blank. Absorbance of all samples were recorded. The concentration of total phenolics was calculated using the equation of the gallic acid standard curve prepared with doses ranged from zero to 500 mg/L gallic acid.

###  Statistical analysis

 All data were analyzed through SPSS (SPSS^®^ software, version 21) and Microsoft Excel. As the results obtained were parametric, a two tailed student paired t-test was performed to determine the differences in BP, PWV and distance ran before and after supplementation. The urine sample concentrations were calculated using the equation of the standard calibration curves on Excel for each test. A 2-way independent t-test was performed to analyze any changes in antioxidants, total phenolic and lipid peroxidation between the intervention and placebo group. The significance of all results was measured against *P* value ≤ 0.05.

## Results and Discussion

 Our 2-weeks study has highlighted some of the beneficial effects of turmeric supplements in healthy volunteers. The novelty of our work is that we have shown that in healthy volunteers, 2-weeks intake of turmeric was able to reduce the expected BP rise in response to exercise followed by a decrease in final BP. An improvement in arterial compliance, lipid peroxidation and oxidative status. In comparison to previous studies, the present study also confirms the antioxidant and anti-inflammatory properties of turmeric as evidenced in the significant increase in antioxidant concentrations (FRAP and total poly-phenols) in the urine samples of the turmeric group.^35,48,49^

###  Baseline characteristics

 Mean BMI for the placebo group was 24.7 ± 3.8, falling between the normal BMI ranges which is 18.5-24.9 kg/m^2^ and few participants were overweight according to WHO guidelines published in 2015.^50^ In comparison, the mean BMI for the curcumin group was slightly higher at 25.8 ± 5.3, crossing into the over-weight category (Table 1). Over 60% of participants reported moderate physical activity two or three times a week. Seven participants had low alcohol intake and all remaining participants (n = 15) were abstainers. There were no significant differences in all baseline parameters between the placebo and the curcumin groups (*P* > 0.05).

**Table 1:** Baseline demographics of all participants participated in the curcumin and placebo groups

	**Placebo** **(n=10)**	**Curcumin** **(n=12)**	***P*** ** value**
Age	21.8 ± 2.2	22.1 ± 1.7	0.729
BMI (kg/m^2^)	24.7 ± 3.8	25.8 ± 5.3	0.524
Caffeine intake (cups/day)	0.8 ± 1.1	1.2 ± 1.1	0.336
Baseline PWV(m/s)	7.49 ± 0.6	7.51 ± 0.9	0.564
Exercise (h/wk)	2.6 ± 1.3	1.8 ± 1.6	0.452
SBP (mm Hg)	120.7 ± 12.4	123.4 ± 15.2	0.871
DBP (mm Hg)	73.9 ± 7.5	75.5 ± 6.9	0.682

Data presented as (mean ± SD). SD: Standard Deviation.

 Smokers were excluded from the study to prevent a modifiable potential risk factor which may influence CVD parameters.^51^ BMI may also have impacted our results and it may be reasonable to investigate the effect of BMI and recruit subjects within a healthy BMI range of 18.5-24.9 kg/m^2^ (WHO 2012) and overweight group.^52,53^ The study implemented a parallel design due to the notion that both crossover and parallel designs offer ad-vantages and disadvantages though parallel trials might still offer ethical and statistical advantages over crossover trials.^54^

###  Cardiovascular parameters

**Figure 2:**
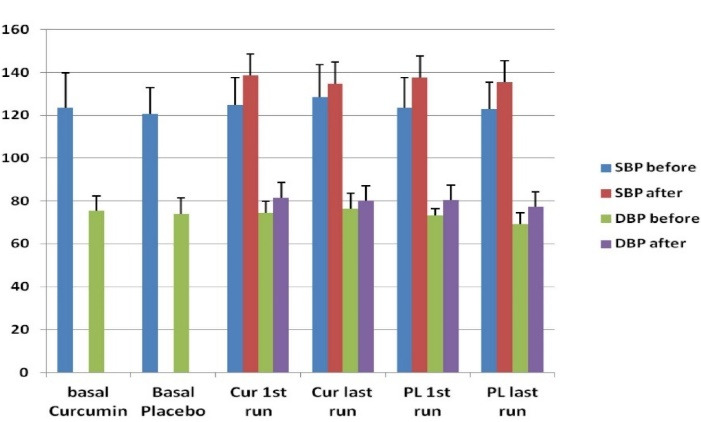
Effect of turmeric intake on Blood pressure. Blood pressure (SBP and DBP) changes between groups. Data presented as (mean ± SD mm Hg). No significant difference in Basal SBP, and DBP between the turmeric and placebo groups. In the first run, turmeric group SBP and DBP increased (*P* = 0.002 and *P* = 0.008), and in the last run, the increase was not significant. Placebo group SBP and DBP increased significantly in the first run (*P* < 0.001) and last run (*P* = 0.008 and *P* < 0.05 respectively)

 There was no significant difference between Basal SBP, DBP and PWV_cf_ in participants taking the placebo or the curcumin (Table 1). After their first run (pre- intervention), there was an expected increase in SBP and DBP in both groups following the 30 min run (Figure 2). In the curcumin group, SBP increased from 124.9 ± 11.8 to 139.8 ± 14.5 mm Hg (*P* = 0.002) and DBP from 74.5 ± 5.4 to 81.8 ± 6.0 mm Hg (*P* = 0.008). In the placebo group, SBP increased from 123.6 ± 14.2 to 138.1 ± 12.5 mm Hg (*P* < 0.001) and DBP from 73.4 ± 3.1 to 80.6 ± 5.1 mm Hg (*P* = 0.001) (see Table 2). In the final run following the intervention, there was also an increase in SBP and DBP of subjects in the curcumin group, but such increase was blunted and not significant compared to pre-exercise values (*P* = 0.111 and *P* = 0.348, respectively) (see Figure 2 and Table 2). However, in the placebo group there was a significant increase in SBP and DBP indicating no effect for the placebo (SBP increased from 123.5 ± 13.0 to 137.3 ± 11.6 mm Hg, *P* = 0.008 and DBP from 70.1 ± 7.8 to 78.1 ± 6.2 mm Hg, *P* < 0.05). The differences in BP between pre-exercise and post exercise (Δ change) were calculated and the results shown in Table 2 as delta change between pre to post run exercise. As shown, turmeric extract rich in curcumin produced a significant reduction in Δ change of exercise induced SBP and DBP (*P* < 0.05) compared to that obtained after the first run. There was no significant change in Δ change of both SBP and DBP in those taking the placebo. No significant change between basal SBP and DBP compared to detections on day 11 in either group was observed, however, the final SBP and DBP in the turmeric group dropped slightly when compared to baseline measurements from 121.8 ± 12.0 to 120.3 ± 14.5 and 75.5 ± 6.9 to 72.6 ± 8.1 mm Hg respectively. Arterial compliance as measured by PWV_cf_ was determined before intervention and on the final day 11 of the study; there was a significant decrease in PWV in the turmeric group (from 7.26 ± 0.97 to 6.7 ± 0.77m/s, *P* = 0.033). However, there was a slight decrease in PWV_cf_ in the placebo group, but it was not significant (from 7.68 ± 0.94 to 7.45 ± 0.52 m/s, *P* = 0.431).

**Table 2:** SPB and DBP detections recorded for all participants at basal, first run and last run before and after the exercise.

**Turmeric**	**SBP**			**DBP**		
	**Pre Exer**	**Post Exer**	**Δ Change**	**Pre Exer**	**Post Exer**	**Δ Change**
Basal	121.8 ± 12.0	-		75.5 ± 6.9	-	
First run	124.7 ± 11.8	139.8 ± 14.5**	15.5 ± 9.6	74.5 ± 5.4	81.8 ± 6.0**	7.3 ± 7.2
Last run	126.4 ± 13.5	133.7 ± 15.3*	7.3±7.0*	76.3 ± 8.6	78.6 ± 7.2^NS^	2.3±6.9*
Placebo	**Pre Exer**	**Post Exer**	-	**Pre Exer**	**Post Exer**	-
Basal	120.6 ± 12.2	-	-	73.9 ± 7.5	-	-
First run	123.6 ± 14.2	138.1 ± 12.5***	14.5 ± 6.1	73.4 ± 3.1	80.6 ± 5.1***	7.2 ± 4.8
Last run	123.5 ± 13.0	137.3 ± 11.6**	13.8 ± 4.3^NS^	70.13 ± 7.8	78.1 ± 6.2*	8.0 ± 6.9^NS^

Note: It also shows the Δ change in SBP and DBP between pre-exercise and post exercise. Significance levels: * *P* < 0.05; ** P < 0.01; *** *P* < 0.001; NS > 0.05

 Our results have shown a significant increase in BP following the 30 min exercise in both groups after the first run which was expected due to the oxidative stress caused by the exercise.^54^ However, following the final run, the exercise-induced increase in BP was only significant in those who received the placebo. The BP increase of turmeric group subjects due to exercise was attenuated and not statistically significant. Moreover, a reduction was observed in SBP and DBP detections in final BP measurements and they were lower compared to baseline values. This supports the data of published studies that showed turmeric intake was able to reduce BP.^55,56^ Other CVD parameters that may be of relevance; Arterial stiffness compliance as measured by PWV_cf_ was significantly reduced after turmeric intake indicating its cardiovascular beneficial effects.^57-59^

###  Urinary biomarkers assays

####  Urinary polyphenols levels

 The concentrations of urinary exertion of polyphenols were compared between both groups using an independent t-test after the first and final runs. The results are shown in Table 3 which indicates that there was a significant increase in the 24 hours exertion of polyphenols following the intake of turmeric supplements only compared to basal values (*P* = 0.022). No significant differences in polyphenols levels were obtained between the basal of the 2 groups and after the 2 runs of the placebo group (*P* > 0.05).

**Table 3:** Total polyphenol concentration of 24 hours urine samples measured in GAE/day

	**Intervention**	**Polyphenol Concentration (mg GAE/day)**	***P*** ** value**
Basal	Placebo	293.3 ± 73.3	-
	Turmeric	276.3 ± 92.2	0.275
First run (pre-intervention)	Placebo	304.8 ± 95.2	0.659
	Turmeric	282.7 ± 85.7	0.772
Last-run (post-intervention)	Placebo	318.4 ± 57.1	0.254
	Turmeric	405.9 ± 132.6	**0.022**

Data presented as (mean ± SD). Significance of data is measured against the basal concentration of each group (*P* value ≤ 0.05).

####  Urinary FRAP analysis

 A similar trend of the FRAP data were obtained to those of polyphenols exertion (Table 4). Results obtained by an independent t-test showed that the difference in antioxidant concentrations be-tween both groups in basal, pre-intervention and post-intervention urine samples were insignificant after the first exercise run. However, there was an increase of urinary antioxidant capacity in the turmeric group post intervention in comparison to the basal value (*P* = 0.031) with mean values increasing from 3.43 ± 1.22 to 3.75 ± 0.94 mmole Fe^+2^/day. This indicates that the turmeric supplements have increased antioxidant levels in the participants group. Other studies have confirmed that polyphenols possess antioxidant and free radical scavenging properties, which reduce low-density lipoprotein oxidation.^60^ We believe that the increase in antioxidant and polyphenol concentrations might have provided a protective mechanism against vascular dysfunction by neutralizing free radicals and reducing BP during exercise.^61^ Thus, the normotensive participants taking turmeric displayed a decrease in BP measurements from baseline to final. In addition, polyphenols are believed to interact with the pathway responsible for the generation of NO from vascular endothelium.^62^ and NO possesses the ability to exert a relaxant effect on the vasculature.^63^

**Table 4:** Mean of antioxidant concentrations of urine samples obtained from the FRAP assay

	**Intervention**	**Antioxidant Concentration** **(mmol Fe**^+2^**/ day)**	***P*** ** value**
Basal	Placebo	3.04 ± 0.57	-
	Turmeric	2.81 ± 1.8	0.216
First run (pre-intervention)	Placebo	3.05 ± 0.42	0.956
	Turmeric	3.12 ± 1.29	0.558
Last-run (post-intervention)	Placebo	3.43 ± 1.22	0.839
	Turmeric	3.75 ± 0.94	0.031

Data presented as (mean ± SD). Significant difference is measured against the basal concentration of each group (*P* ≤ 0.05).

####  Urinary TBARS results

 Data presented in Table 5 shows that the basal concentration of MDA (μmole/day) urinary levels in both groups were not significant (*P*> 0.05). As expected, due to the exercise-induced oxidative stress, there was an increase in MDA concentration in the curcumin and the placebo groups after the first run (*P* = 0.026 and *P* = 0.002 respectively). At the final run, there was also a significant increase of MDA concentration in the placebo group only (*P* = 0.04), however in the curcumin group, there was a small increase in MDA concentration which was not significant (*P* = 0.328). This could be due to the anti-inflammatory and antioxidant effects of turmeric.Further evidence of turmeric potential effects on CVD risk factors was that turmeric extract intake has attenuated the exercise-induced increase of lipid peroxidation (assessed as MDA by the TBARS assay) compared to those on the placebo. Moreover, turmeric did display other beneficial effects in our participants, for example, 3 subjects in the turmeric group reported no signs of DOMS after their last run (post intervention). We know that DOMS occurs due to inflammation and the activation of NF-kB and therefore, we can suggest that turmeric may possess muscle protective properties.^64^ However, as inflammatory markers were not analyzed in this current study, future studies might investigate the effects of turmeric or curcumin on inflammatory markers and other mechanisms that might be involved.

**Table 5:** Results of Lipid peroxidation assessed as urinary MDA levels in all subjects of the study

	**Intervention**	**MDA Concentration** **(μmol / day)**	***P*** ** value**
Basal	Placebo	2.263 ± 0.74	-
	Turmeric	2.249 ± 0.91	0.825
First Run (Pre-Intervention)	Placebo	3.422 ± 0.97	0.026
	Turmeric	3.472 ± 1.2	0.002
Last-Run (Post-Intervention)	Placebo	3.627 ± 1.43	0.040
	Turmeric	2.662 ± 0.68	**0.328**

Note: Data is presented as (mean ± SD). Significant differences are measured against the basal concentration of each group.

###  Physical activity 

 Mean results from the Borg scale of perceived exertion indicated that the turmeric group participants were able to run at a higher intensity after the administration of supplements as opposed to before with lower efforts. Their level of exertion decreased from 5.08 ± 1.38 to 4.51 ± 1.1 but no significance was obtained (*P* > 0.05). However, the distance ran by the participants taking turmeric extract in the last run was significantly longer (*P* = 0.005) compared to that in the first run as shown in Table 6. In comparison, the placebo group participants ran at a lower rate post-intervention compared to those taking turmeric, and the distance and Borg scale recorded after the final run were not significant (*P* > 0.05). Table 6 shows mean exercise and perceived exertion parameters of participants before and after the intervention. Significant difference in the last run is measured against that in first run (*P* ≤ 0.05). Exercise performance following the turmeric intake showed a slight but significant increase in the distance achieved during the 30 minutes run compared to those taking the placebo. In addition, Borg score of perceived exertion was lowered and thus the turmeric group felt they were able to run at a greater intensity during the last run-in comparison to their first run.^65^ Williams 2008 has examined the effects of running on the prevalence of high BP and suggested a potential link between the two, reporting that running at a great intensity for a long duration can result in hypertension.^66^

**Table 6:** Mean exercise and perceived exertion parameters of participants before and after the intervention

	**First Run**	**Last Run**	***P*** ** value**
Curcumin			
Distance (km)	3.66 ± 0.81	3.97 ± 0.94	**0.005**
Speed (km/h)	7.32 ± 1.61	7.45 ± 1.99	0.227
Borg Scale	4.08 ± 1.38	3.51 ± 1.1	0.131
Placebo			
Distance (km)	3.38 ± 0.84	3.66 ± 0.72	0.186
Speed (km/h)	6.85 ± 1.67	7.22 ± 1.44	0.219
Borg Scale	4.13 ± 1.69	4.06 ± 0.95	0.924

Note: Data presented as (mean ± SD). Significant difference in the last run is measured against that in first run (*P* ≤ 0.05).

###  Dietary intake assessment 

 Data presented in Table 7 which shows a slight increase in mean total energy and fat after the administration of supplements in the turmeric group compared to pre-intervention intake, but it was not significant. Statistical analysis through an independent t-test verifies an increase in energy and protein intake by the placebo participants before intervention compared to the curcumin group, suggesting that the turmeric supplements have no effect on total energy intake. Results from the health status questionnaire showed that the weight and height (BMI) of the 2 groups were not significantly different. However, there were 4 volunteers of the turmeric participants had a BMI of 30 kg/m^2^ which might have hindered their physical activity.^67,68^ Obesity is generally associated with the presence of oxidative stress through inflammation, and it is justified to suggest that the high BMI results of certain participants may have resulted in increased levels of exercise induced oxidative stress.^69,70^

**Table 7:** Dietary daily intake values of all participants for turmeric and placebo groups

	**Pre-intervention**	**Post-intervention**	***P*** ** value**
Daily intake turmeric			
Total energy (kJ)	5465.2 ± 2451.4	5827 ± 2566.5	0.611
Fat (g)	47.6 ± 25.4	58.9 ± 21.7	0.119
Protein (g)	50.1 ± 24.9	48.3 ± 23.6	0.775
CHO (g)	172.9 ± 79.6	173.1 ± 107	0.994
Daily intake placebo			
Total energy (kJ)	6162.2 ± 2269.4	5996.8 ± 1972.5	0.201
Fat (g)	47.6 ± 19.4	61.1 ± 17.7	0.619
Protein (g)	60.1 ± 21.9	54.3 ± 20.6	0.275
CHO (g)	206.9 ± 91.6	194.1 ± 138.4	0.124

Note: Data presented as (mean ± SD). Significance tests were performed between pre- and post-interventions.

 It would be worthwhile to mention some limitations in this present study, which may have influenced the results. The study was of short duration with low number of participants. It was also unclear whether all participants were compliant in completing the full eight days course of supplements provided which might altered some of our results. Indeed, further investigation would be required to determine the effects of turmeric and/or curcumin on oxidative stress and CVD risk factors using a larger sample size to minimize errors. Finally, the influence of oral bioavailability of turmeric should be considered in relation to gut microbiota of individuals. Many studies have exposed the poor absorption of turmeric in the gastrointestinal tract due to its poor solubility in water. As turmeric possesses many desirable properties, numerous approaches have been undertaken to find a solution for this disadvantage.

## Conclusion

 Our study has demonstrated that turmeric possesses antioxidant and anti-inflammatory properties which have proven to lower BP and improve some cardiovascular risk factors. Turmeric intake was also shown to improve exercise performance and ameliorates oxidative stress. It also seems evident that the consumption of the ancient yellow spice seemed to decrease exercise induced SBP, DBP and lipid peroxidation. Larger studies investigating the effects of turmeric extract on oxidative stress, exercise performance and other cardiovascular parameters would be warranted.

## Acknowledgments

 The authors would like to acknowledge the technical support of Anum Mizher of Queen Margaret University. We are grateful for all the volunteers who participated in this research study. The authors are pleased to acknowledge the cooperation and support of Al-Ahliyya Amman University, Amman, Jordan in the publication of this manuscript.

## Competing Interests

 The authors declare no conflicts of interest.

## Ethical Approval

 The study was conducted in accordance with the Declaration of Helsinki and approved by the Institutional Review Board of Queen Margaret University (code Honors/11010149/Curcumin/BSc-NUT/DNBS/QMU).
